# Inflammatory mediators and the RAGE pathway in placental tissues of pregnancies complicated by severe preeclampsia

**DOI:** 10.3389/frph.2025.1587699

**Published:** 2025-08-11

**Authors:** Neelima Chandra, Thomas D. Kimble, Kathleen R. Heim, Sharon M. Anderson, Andrew P. Wong, Andrea R. Thurman, Gustavo F. Doncel

**Affiliations:** ^1^ CONRAD, Eastern Virginia Medical School, Old Dominion University, Norfolk, VA, United States; ^2^ Department of Obstetrics and Gynecology, Eastern Virginia Medical School, Norfolk, VA, United States; ^3^ VHC Health Physicians, Charlotte S. Benjamin Center for Women’s Health, Arlington, VA, United States

**Keywords:** calcium binding protein (S-100), cyclooxygenase 2 (COX-2), high mobility group box protein (HMGB1), interleukin 8 (IL-8), nuclear factor kappa B p65 sub unit (NF-kBp65), receptors of advanced glycation end products (RAGE)

## Abstract

**Introduction:**

Preeclampsia (PE) is a complex multisystem disorder of pregnancy associated with abnormal placentation, vascular anomalies, and systemic inflammation and hypertension. Previous research assessing inflammatory triggers of the condition used plasma, amniotic fluid, or explant samples. Studies using placental tissue from either vaginal or cesarean deliveries are confined to semiquantitative analysis using subjective scoring methods and generally involve a small sample size.

**Methods:**

In this study, we have quantified the expression of inflammatory mediators by immunohistochemical image analysis of archived placental tissues obtained from cesarean delivery of preeclamptic, chorioamnionitic, and normal pregnancies.

**Results:**

Among the inflammatory mediators, we found a significant elevation in the expression of receptors of advanced glycation end products (RAGE) and two of its damage-associated molecular pattern proteins (DAMPs) and ligands, the high mobility group box protein HMGB1 and the calcium binding protein S100, in preeclamptic tissues as compared to normal placentas. In addition, we observed a significant increase in the master pro-inflammatory transcription factor, nuclear factor kappa B p65 subunit (NFκB), as well as non-significant increases in cyclooxygenase 2 (COX-2) and interleukin 8 (IL-8) in the PE group.

**Conclusion:**

This study provides insight into the relationship of tissue inflammatory mediators with severe preeclampsia and the RAGE associated signaling complex, suggesting a pathogenic role for this pathway which has clinical implications for the understanding, diagnosis, and potential novel therapeutic approaches to the syndrome.

## 1 Introduction

Preeclampsia (PE) is a pregnancy-associated condition occurring at 20 weeks of gestation or greater and is traditionally diagnosed when a pregnant woman presents with increased blood pressure, edema, and proteinuria (1). PE affects 5%–8% of pregnancies, with higher risk factors associated with primigravida, older age, diabetes, hypertension, obesity, and antiphospholipid syndrome, among others (2, 3). Although the exact cause of PE is not known, the condition seems to develop when the trophoblast fails to invade the decidual arteries, thereby causing abnormal placental development and potentially leading to an interruption of sufficient nutrient transport (4, 5). PE may lead to long-term health complications of the fetus and mother (6) or, more immediately in severe cases, preterm birth, neonatal morbidity, and mortality. PE is a highly variable syndrome with multiple clinical presentations and emerging treatment strategies (7, 8), and the understanding of the etiology of this state of abnormal placental development and function is operatively incomplete. Inflammatory markers have proven a promising avenue as predictors of PE (9, 10), as there have been several reports of uncontrolled inflammation in individuals suffering the syndrome (11, 12). Local and systemic inflammation arising from a chronic state of runaway immune activation has been identified as a common pathological change associated with this condition (11, 13, 14) but the exact mechanisms that mediate the complex hierarchy of risk factors, epigenetic changes, and signaling pathways that lead to the development of PE remain unclear.

In the present paper, we describe and compare markers of inflammation, immunohistochemically identified in placentas from normal healthy term pregnancies (negative control) and those complicated by chorioamnionitis (positive control) and PE with severe features (experimental group). Chorioamnionitis is a broad spectrum of disease characterized by inflammation or infection during pregnancy in intrauterine structures like the placenta (15), leading to serious pregnancy complications and long term adverse outcomes (16). Chorioamnionitis is specifically associated with a cascade of inflammatory cytokines during periods of cellular stress (17) and, keeping in mind the known inflammatory status of this condition, it has been used as an appropriate positive control for examining relative expression of inflammatory markers in preeclamptic placental tissue.

All placental tissues were obtained from Cesarean section derived human placentas to avoid the confounding impact of labor, since labor has been associated with tissue inflammatory changes (18). Placentas were obtained in the third trimester at the time of indicated Cesarean section, prior to labor. The specific goal of this study was to investigate differential expressions of receptors of advanced glycation end product (RAGE), its ligands high mobility group box protein (HMGB1) and calcium binding protein (S100A10), as well as the inflammatory mediators nuclear factor kappa B p65 subunit (NF*κ*B), cyclooxygenase 2 (COX-2), and interleukin 8 (IL-8), at the placental tissue level in pregnancies associated with PE in comparison to normal pregnancies and those associated with a well-known inflammatory condition, chorioamnionitis. HMGB1 and S100A10, as damage associated molecular pattern proteins (DAMPs), resulting from extracellular host responses to microbial pathogens (19–21), were of particular note for investigation due to their role as “danger signals” activating RAGE (22, 23) and the following sustained activation of NFκB, which can recruit inflammatory cells causing tissue damage. The association of heightened expression of these markers in placentas from pregnant women with PE has pathogenic and potentially diagnostic implications.

RAGE plays a multifaceted role in the placenta, impacting both normal placental function and contributing to pregnancy complications (24). It acts as a receptor for various ligands, including advanced glycation end products and other inflammatory molecules, and its activation can trigger inflammatory and stress responses within the placenta. HMGB1 is a protein with dual roles in the placenta, acting as both a nuclear protein and a pro-inflammatory cytokine when released extracellularly, and is involved in early pregnancy events like embryo implantation and uterine decidualization where elevated levels of HMGB1 are associated with various pregnancy complications (25). The HMGB1–RAGE pathway is strongly associated with trophoblast damage, systemic inflammation, and the maternal endothelial pathology seen in preeclampsia (25). Similarly, S100 proteins play a role in human placenta development, influencing trophoblast proliferation, motility, and syncytialization, and elevated or altered levels of S100 proteins have been observed in women with preeclampsia, both in placental tissue and bodily fluids (26). The S100-RAGE axis likely plays a role in normal placental development and the immune response to pathogens, maintaining a healthy pregnancy. Activation of RAGE, especially by DAMPs like certain S100 proteins, can trigger inflammatory signaling pathways, including the NF-κB pathway, which leads to the release of proinflammatory cytokines (27). The HMGB1–RAGE and S100–RAGE axes play pivotal roles in preeclampsia by mediating placental and systemic inflammation, endothelial dysfunction, and anti-angiogenic pathways.

## 2 Materials and methods

Archived paraffin embedded placental tissues were obtained from the Department of Pathology at Sentara Norfolk General Hospital (Norfolk, VA) or Sentara Leigh Memorial Hospital (Norfolk, VA) under an approved Eastern Virginia Medical School (EVMS) IRB # 11-10-WC-0235, supplemented by commercially procured tissues from Capital Biosciences (Gaithesburg, MD, USA) following preestablished selection criteria to expand the sample size. Placentas were from women undergoing elective or indicated Cesarean section at term, prior to labor.

We have used the American College of Obstetricians and Gynecologists (ACOG) definition of PE to identify women with this pregnancy complication (28). Selection criteria included placental tissues from normal pregnancies termed as the negative control group (*n* = 20), placental tissues from pregnancies complicated by chorioamnionitis, termed as the positive control group (*n* = 20), and placental tissues from pregnancies complicated by PE termed as the study group (*n* = 20). Pregnancies did not show clinical signs of labor at delivery and babies and placentas were delivered by Cesarean section in the third trimester. We excluded women who developed preterm labor in the current pregnancy, any use of a COX inhibitors (e.g., non-steroidal anti-inflammatory drugs, NSAIDs) in the past 72 h, sexually transmitted infection during the pregnancy, a lower genital tract infection within 14 days of delivery, active HIV or herpes simplex virus type 2, uncontrolled diabetes, smoking, current HELLP syndrome, or pre-existing hypertensive disorders.

### 2.1 Immunohistochemical (IHC) staining

Paraffin embedded placental tissues were cut to 5–6 micron sections and placed on slides for IHC staining. The slides were processed for staining as previously described (29). In brief, slides were deparaffinised, dehydrated, and finally rehydrated. The slides were then placed in an antigen retrieval solution bath (citrate buffer at pH 6.2, Dako) and maintained at high temperature. This was followed by subsequent washings with phosphate-buffered saline (PBS, pH 7.4) and treatment with specific protein block. After subsequent washing with PBS, the tissues were treated with specific serum protein for 30 min to block nonspecific binding. The tissues were then treated with primary antibodies COX-2, IL-8, NF*κ*B, HMGB1, RAGE, and S100A10. Source and dilutions of the specific antibodies are listed in Table 1. The slides were placed at 4°C overnight followed by biotinylated secondary antibody treatment on the next day (Table 1). Avidin: biotinylated enzyme complex (ABC reagent), from Vector labs (Burlingame, CA) was applied on the slides. The tissues were finally treated with AEC chromogen–substrate (SkyTek Labs, Mississauga, Ontario, Canada) and viewed under a Nikon E800 microscope. Slides were then mounted with Accergyl mounting media (Accurate Chemicals, NY) and cover slipped. Images were captured using a CCD camera (Spot Camera, Diagnostic Instruments MI, USA).

**Table 1:** The primary and secondary antibodies used to identify target molecules in placental tissues are listed along with their source and dilutions. Other reagents for IHC staining are given as a footnote.

Antibody	Source	Cat. #	Dilution
Primary			
COX-2	Abcam	ab15191	1:250
IL-8/CXCL8	R&D	AF-208-NA	10 ug/ml
Nfkβ p65	Millipore	MAB3026	1:100
HMGB1	Abcam	ab79823	1:500
RAGE	Santa Cruz	sc5563	1:50
S100A10	Santa Cruz	sc50450	1:50
Secondary			
Anti-mouse in horse	Vector	BA-2000	1:200
Anti-rabbit in goat	Vector	BA-1000	1:200
Protein block			
Normal horse serum	Vector	S-2000	1.5%
Normal goat serum	Vector	S-1000	1.5%

Antigen retrieval solution pH 6.2 (Dako. S1699).

ABC KIT (Vector Labs) cat# PK 6100.

AEC substrate (# ACE 500) & AEC Chromogen (#ACD015) from SKYTEK lab.

Accergyl mounting media (# AXL 686) from Accurate Chemical.

### 2.2 Image analysis and statistics

The immuno-labelling of the proteins was analyzed employing ImageJ software (NIH, Bethesda, USA). In brief, several areas within a stained slide were randomly selected using a Nikon 800 microscope. Images were captured using CCD Camera (Spot Camera, Diagnostic Instruments, MI) at 400× magnification. The integrated optical density (IOD) of the positive stain on the villi of the placenta was analyzed using ImageJ. Total villi analyzed per tissue section were ∼50 (from *n* = 20/group specimens). The IOD values of the control slides (with no primary antibodies) were subtracted from the IOD values for each tissue (29, 30). Values were calculated as Mean ± SD of *n* = 20/group. Statistical analysis was performed using ANOVA w/Dunnett's multiple comparison test (using GraphPad Prism version 6.00 for Windows, GraphPad software, La Jolla, California, USA). The PE group was compared to both positive and negative controls and significance tested at *p* < 0.05. A-priori power analysis of our expected effect size, which observationally exceeded 0.5 for multiple biomarkers of interest, confirmed suitability of our sample size for each study group (*n* = 20) with a type I error probability of 0.05 and a minimum power of 0.8. Ultimately, the power of our study exceeded 0.8 and was around 0.83 at the analyzed sample sizes.

## 3 Results

**Figure 1:**
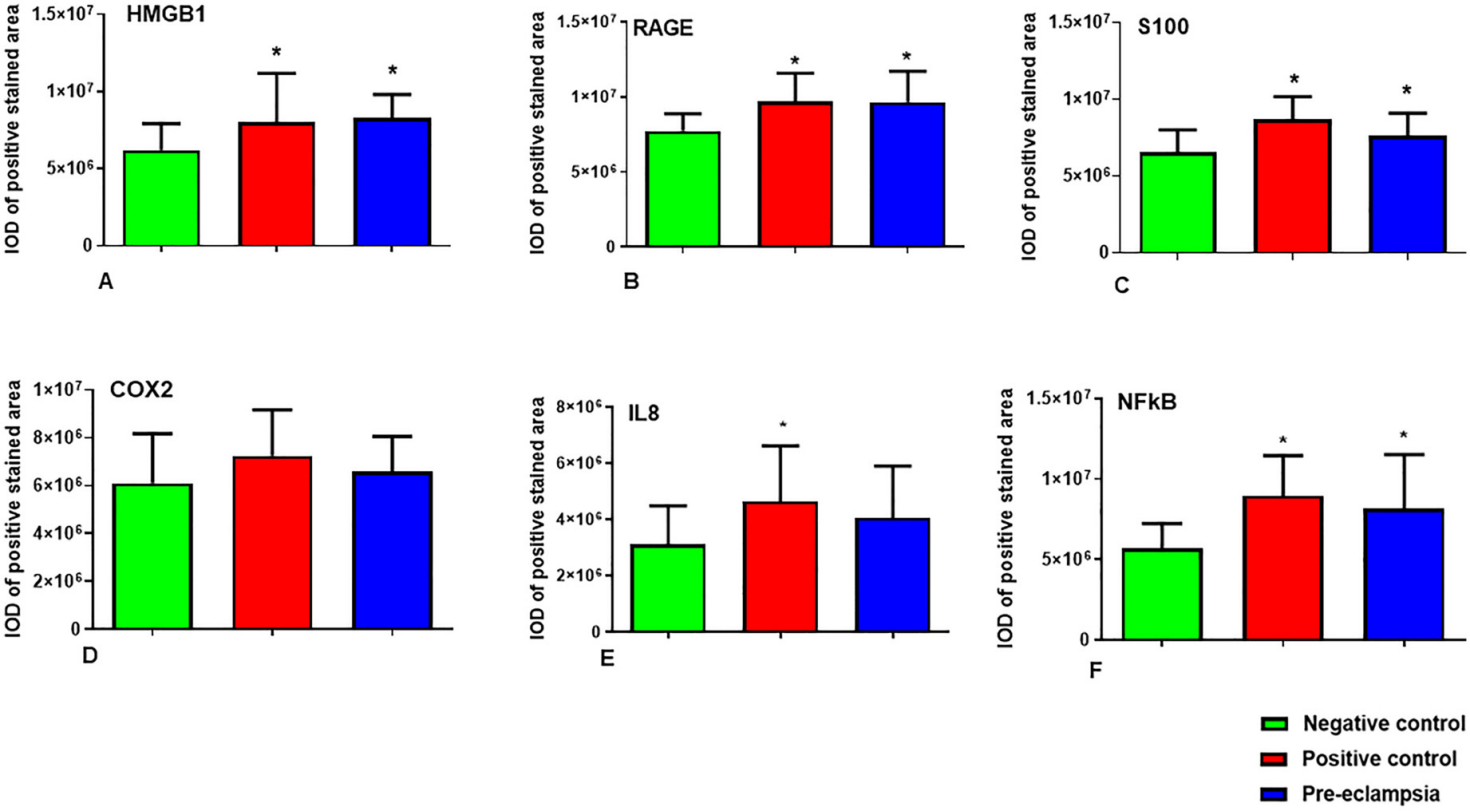
**(A–F)**: Graphs show integrated optical density (IOD) values of the stained villi employing imageJ analysis for **(A)** HMGBI **(B)** RAGE **(C)** S100A10 **(D)** COX-2 **(E)** IL-8 and **(F)** nFΚB. Values are mean ± SD for *n* = 20, each for negative & positive controls and the PE group. Statistics were done using ANOVA supported with Dunnett's multiple comparison test. The data set can be found within Supplementary Table S1. *denotes significant increase in IOD of proteins, as compared to the negative control group.

**Figure 2:**
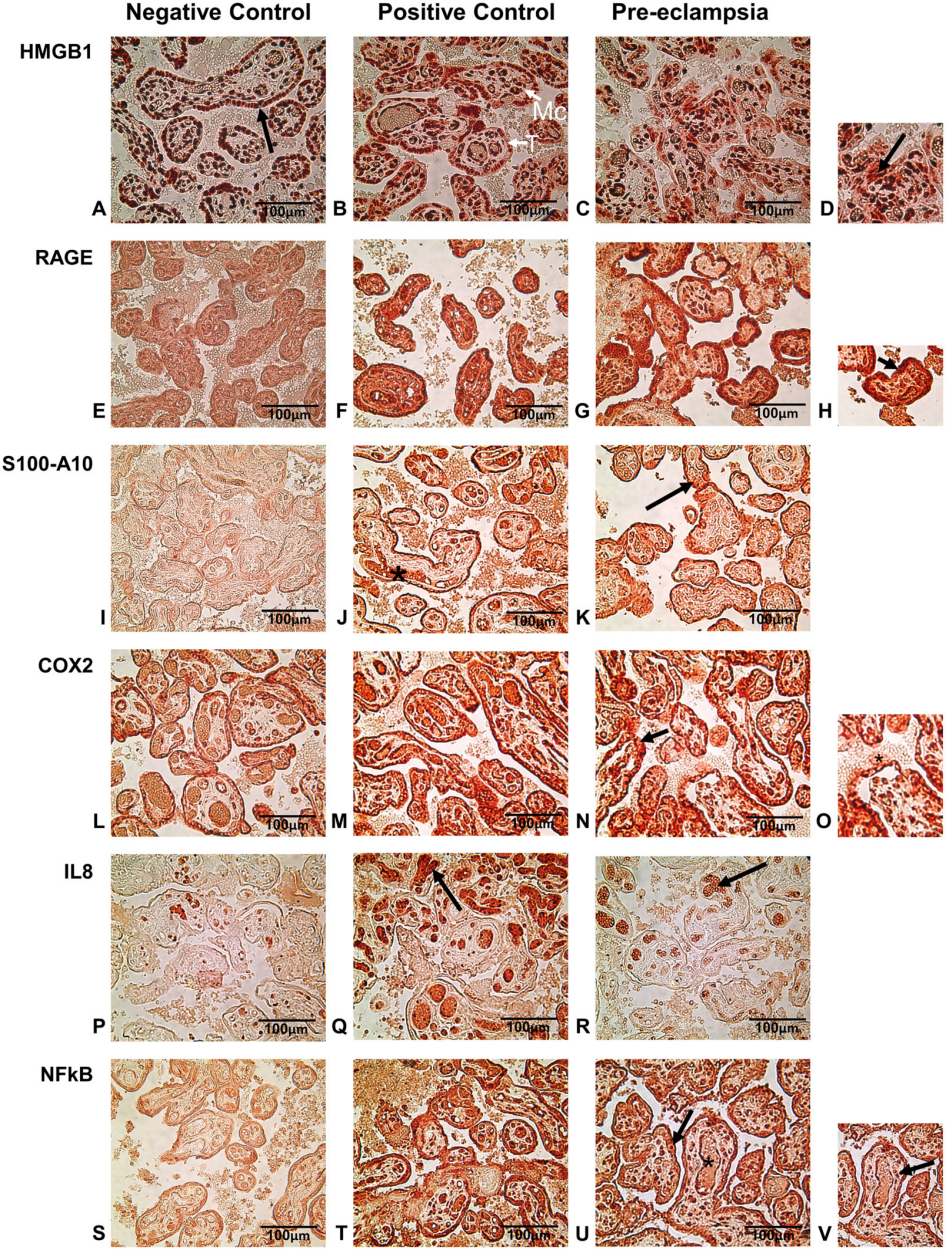
**(A–D)** immuno-histochemical staining of HMGB1 in nucleus (arrow in **A**) of negative, positive and preeclamptic placental tissues. **(D)** Enlarged picture of a section of PE tissue. The arrow shows extranuclear release of HMBG1. **(E–H)** Immunolocalization of RAGE was mainly in the syncytiotrophoblast and in the cytoplasm. Arrow in the enlarged picture **(H)** shows intense staining in syncytiotrophoblasts of PE and positive control tissues. **(I–K)** S100A10 staining showed strong expression of this protein in fetal blood vessel (*) in positive control placenta and chorionic villi (arrow) both in positive control and PE group tissues. **(L–O)** Strong COX-2 staining of syncytiotrophoblast (arrow) and release in maternal blood (* in enlarged insert-**O**) in positive control and PE placentas. **(P–R)** IL-8 staining by IHC showed the expression in fetal blood vessels within the villi (arrow) mostly in positive control tissues and to a lesser extent in PE placentas. **(S–V)** Immuno-histochemical staining for NFκB showed strong localization mainly in trophoblast and mesenchymal cells in positive control and PE tissues. Some expression of NF*κ*B was also observed in fetal blood vessels in these groups. A few cells in the syncytiotrophoblast were enlarged in PE placenta (arrow in **V**) with a large nucleus. T, Trophobast; Mc, mesenchymal cells. All pictures were taken at 400× magnification.

HMGB1 staining was observed in the nucleus of all the tissues by IHC. HMGB1 expression was detected in the cell nucleus of chorionic villi including syncytiotrophoblast and also in the nucleus of the cells in connective tissues. Analysis of these villi showed significantly higher expression of HMGB1 in PE placental tissues as compared to those of the normal (negative control) group. The intensity of the staining in the PE group was comparable to that of the chorioamnionitis (positive control) group (Figures 1A, 2A–C). In general, there was an extranuclear localization of HMGB1 in the surrounding cells of the placenta, with the PE group registering higher staining, especially in the cells of syncytiotrophoblast (see inset in Figure 2D).

Tissue staining for RAGE was mostly observed in the cells of the trophoblast and cells within chorionic villi (connective tissue). The staining was mostly restricted to the cytoplasm of the cells, which showed a higher expression of RAGE in the PE groups as well as in the positive control with chorioamnionitis. Few nuclei of the syncytiotrophoblast also showed staining in the PE and chorioamnionitis groups. RAGE expression was also profound in the cytoplasm of mesenchymal cells in both groups. The quantification of the chorionic villi showed statistically higher intensity of RAGE staining in both these groups as compared to the negative control from normal pregnancies (Figures 1B, 2E–H).

A regulator of cell function, S100A10, was detected mostly in the cytoplasm of trophoblast cells in placenta and was also seen around the fetal blood vessels. Expression of S100A10 in the normal placenta was very low (Figure 2I) in contrast to very strong expression in the PE and positive control tissues, both in the trophoblast as well as around the fetal blood vessels and the mesenchymal cells (Figures 2J,K). Quantification of S100A10 expression in the villi showed a statistically higher intensity in chorioamnionitis and PE groups as compared to the normal tissues (Figure 1C).

The IHC staining of COX-2 showed the protein in the cytoplasm of syncytiotrophoblast as well as in the mesenchymal cells of connective tissue within the chorionic villi (Figures 2L–O). This enzyme was also detected around the fetal blood vessels in the positive control groups (Figure 2M). Expression was greater in chorioamnionitic placenta and to a lesser extent in the PE group. However, these changes were not statistically significant in comparison to the normal placenta (Figure 1D).

IL-8 expression was identified in and around the cells in fetal blood vessels within the connective tissues of the villi in the PE group. IL-8 accumulation was very high in the chorioamnionitic placental tissues (Figure 2Q) and its intensity was significantly higher in these tissues than in those of the normal group (Figure 2P). Upon analysis, we found that although the expression of this chemokine was greater in stained placental tissue with PE (Figure 2R), its difference did not reach statistical significance when compared to the normal pregnancy group (Figure 1E).

Compared to the negative control (Figure 2S), positive staining for NFκB by IHC was seen in abundance in the cells of trophoblasts as well as in mesenchymal tissues both in PE and positive control tissues (Figures 2T–V). Expression of NFκB was highest in the cytoplasm of the cells but was also strong within the fetal blood vessels in the chorioamnionitic placenta, with less prevalence in PE and in negative control groups. Image analysis showed significantly higher protein expression both in positive control and PE placentas as compared to the negative control group (Figure 1F).

## 4 Discussion

In this cohort of women who delivered at term, we observed significantly higher amounts of several specific inflammatory markers, mainly those involving the RAGE associated complex including its alarmin ligands, HMGB1 and S100A10, and activated NFκB, at a tissue level in the PE placentas compared to women with normal pregnancies. Unlike the tissues from normal pregnancies matching gestational ages and Cesarean section deliveries, increased expression of these inflammatory mediators was also observed in placentas affected by chorioamnionitis. The tissue inflammatory markers of PE were similar to those of chorioamnionitis, which is a clear example of an acute placental inflammation (31), and it served as a positive control to which the PE inflammatory changes were compared.

HMGB1, a proinflammatory danger signal originally described as a DNA binding protein and transcription factor released extracellularly, has been identified as a key modulator of inflammatory responses (32). An increase in circulating levels of HMGB1 in many inflammation-related diseases, including PE, has been previously reported (33)*.* In addition, Tangerås et al. (34) demonstrated significantly higher serum levels of HMGB1 in both normal and preeclamptic women compared to non-pregnant women. They also reported high expression of HMGB1 and its receptor TLR4 in syncytiotrophoblasts of all placentas; however, cytoplasmic HMGB1 expression was significantly increased in preeclamptic pregnancies specifically complicated by fetal growth restriction (34). We observed a heightened expression of HMGB1 in preeclamptic tissues as compared to those of healthy term pregnancies delivered by Cesarean section. Furthermore, there was a strong nuclear expression of HMGB1 in placentas of the PE group in both trophoblastic and mesenchymal cells in the connective tissue. A similar expression of HMGB1 has been reported in the cytoplasm of the syncytiotrophoblast and trophoblastic debris in a placental explant study with increased protein where the tissues were treated ex vivo with preeclamptic sera (32).

The S100 group of proteins, which are calcium binding proteins, among other functions, are involved in regulation of cellular differentiation, proliferation, apoptosis, and inflammation along with Ca2 + homeostasis (35). These proteins have been shown to be present in amniotic fluid, endometrium tissue, and fetal brain (36). A positive correlation of the S100 group of protein has been seen with inflammatory cytokines, displaying interactions among those inflammatory factors (37). The higher expression of S100A10 in preeclamptic placentas in our study, especially in the trophoblast and some mesenchymal cells, can be correlated to an induction of the RAGE pathway for which S100 proteins are ligands (24, 35). This higher expression in PE tissues was comparable to that identified in the chorioamnionitis positive control placenta.

A multi-ligand cell surface receptor for advanced glycation end products, RAGE, is present on numerous cells including those from the amnion and choriodecidual tissues (38). Its activation mediates cellular injury. RAGE was shown to be expressed in syncytiotrophoblast and cytotrophoblast of normal pregnancy at term (24, 39, 40), possibly related to tissue changes associated with preparation for labor and fetal membrane rupture (41, 42). We observed placental expression of RAGE in all groups, but its expression in PE tissues was significantly higher, suggesting a potential pathogenic role. Apart from its expression in the syncytiotrophoblast, there was strong expression in cells of the connective tissue in chorionic villi. Higher amounts of RAGE were observed in PE placentas as compared to those in the normal pregnancy group. In this regard, there have been conflicting reports on RAGE expression by different authors. Holmund et al. (43), for example, did not see any difference in normal vs. PE placentas at term or near term, but this finding may be confounded by their small sample of placentas derived from elective Cesarean sections. However, several other studies reported increased in sRAGE levels in maternal blood and amniotic fluid from PE patients (39, 44). It has been demonstrated in animal models that under certain stimulation both RAGE and HMGB1 play a fundamental role in inflammation and oxidative stress induced tissue injury (19), mechanisms that have been also described in PE (32, 45).

The damage associated molecular pattern proteins (DAMPs), also known as alarmins, which are the results of host response to microbial pathogens, include HMGB1 and S100 proteins (19–21). Upon activation, these DAMPs are released in excess in extracellular compartments, thereby turning into a “danger signal” that activates RAGE (22, 23). In general, binding of DAMPs to RAGE causes sustained activation of NFκB, thereby recruiting inflammatory cells, which lead to tissue damage. There is strong evidence that maternal immune system activation contributes to the development of PE (46). We found a similar pattern of extracellular release, both for HMGB1 and S100A10 in the preeclamptic placenta, showing high intensity of protein expression as compared to the normal placenta followed by a significant elevation in NFκB expression both in PE and positive control placentas, confirming the presence of inflammatory triggers under PE conditions. NFκB affects several pathways within the placenta that are also found to lead to PE; however it is still unclear how exactly NFκB activation contributes to abnormal placental development and function (6). NFκB is a key regulator of various cellular processes, and its dysregulation in the placenta is strongly implicated in the development of preeclampsia whereby NF-κB signaling in trophoblast cells inhibits proper trophoblast differentiation and invasion (6, 47). Genes involved in cell survival and migration, such as matrix metalloproteinases and tissue inhibitors of metallo-proteinases, which control the extracellular matrix and placental cell invasion, are specifically regulated by NF-κB and the related immune response at the maternal-fetal interface (48). In preeclampsia, altered NF-κB signaling can contribute to an imbalance between pro-inflammatory and anti-inflammatory immune cells, leading to excessive immune activation in the placenta and further exacerbation of the inflammatory response (6). COX-2 has been identified as a mediator in pathophysiologic reactions such as inflammation and is a key enzyme in the biosynthesis of prostaglandins. COX-2 has been reported in placental tissue samples (49) and was shown to increase in the healthy pregnant women in labor at term (50, 51). There have been conflicting results regarding the levels of this enzyme or activities in the placentas of women with PE, which have been found increased (52), unchanged (53), or decreased (54). Studies with decreased COX-2 in PE have been linked to increase in oxidative stress (49). Several other studies have shown that COX-2 plays a vital role in human decidualization and that its decreased expression impairs decidualization and vascularization of the endometrial stroma which may be playing a role in occurrence of PE (55). We identified COX-2 in all the groups of placentas tested, with a slightly higher expression in PE and chorioamnionitis which did not reach statistical significance against the negative control (normal pregnancies). This might be due to individual variation in the groups and/or the need to evaluate a larger sample size.

Interleukin-8 (IL-8), a member of the CXC family of chemokines, has been found to be associated with several systemic inflammatory diseases, including PE (56, 57). Several studies showed higher IL-8 plasma levels in preeclamptic women and increased IL-8 production by maternal peripheral blood mononuclear cells (PBMCs) in PE has been demonstrated (58, 59). Pro-inflammatory cytokines in general are produced by placental trophoblasts, stromal cells and macrophages, and they are also secreted by monocytes. Monocytes may represent an important source of pro-inflammatory cytokines, as maternal PBMC from women with PE produced higher levels of pro-inflammatory cytokines, including IL-8 (60–62). Studies have shown that IL-8 is increased in serum of PE subjects when compared to the healthy controls (63, 64). We found a trend to higher expression in PE and chorioamnionitis placental tissues, not reaching statistical significance against the negative control.

**Figure 3:**
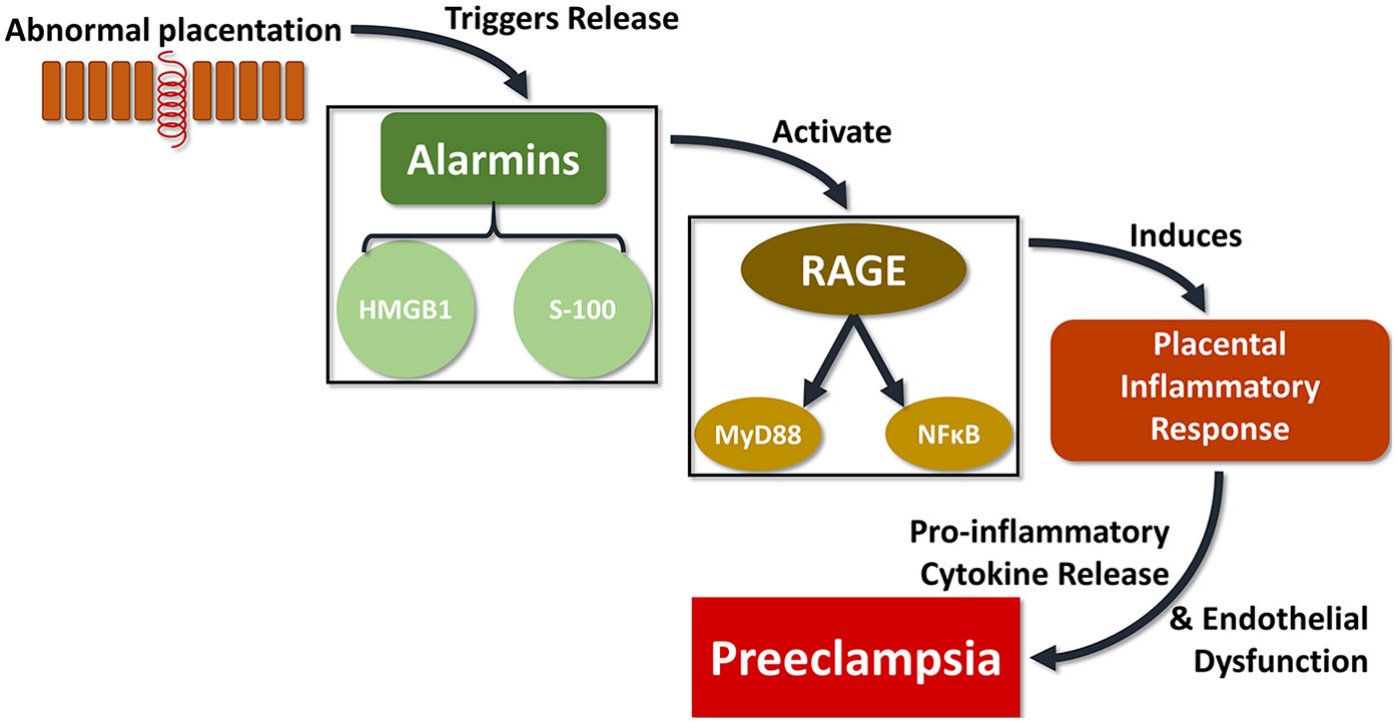
The proposed signaling pathway connecting abnormal placentation events with PE could be critically linked with the RAGE complex. In one possible schema, defects in the placental cytotrophoblast differentiation pathway trigger the release of alarmins such as HMGB1 and S-100, which in turn bind to the multi-ligand receptor RAGE to produce a tissue inflammatory reaction through the release of proinflammatory cytokines induced by MyD88 and NFκB activation, which ultimately results in vascular and tissues alterations leading to PE.

In spite of the existence of several studies on systemic inflammatory markers associated with PE (65–67), the exact pathophysiology of PE remains mostly obscure. These abnormalities appear to be associated with changes in different components of the signaling pathway that mediates cytotrophoblast migration/invasion (68)*.* Additionally, less research has been focused on placental tissue mediators. Detection of inflammatory mediators in placentas themselves of preeclamptic women is important to identifying potential pathogenic mechanisms and potential markers of increased risk for developing the disorder. In the present study, we found that HMGB1, S100A10, RAGE, and NFκB were all significantly increased in placental tissues affected by PE. We postulate that abnormal placentation triggers the release of alarmins, HMGB1 and S-100, which in turn bind to RAGE to induce a tissue inflammatory reaction through the release of proinflammatory cytokines triggered by MyD88 and NFκB activation (Figure 3).

This pathway has been implicated in other tissue inflammatory disorders (69–73), accentuating a potential role for the signaling pathway emerging from the interaction of the multiligand receptor RAGE with its stimulatory ligands ultimately contributing to the pathogenesis of PE. Interestingly, if RAGE were to play a critical role in the development of PE, small molecule inhibitors have been tested in animal models of other inflammatory disorders from diabetes to cancer to Alzheimer's (74–76), and represent a potential novel therapeutic approach for the treatment and prevention of PE (70).

This study has some limitations that warrant consideration. First, due to availability of data or requirements of the IRB, we did not have additional clinical information on hand for the patient population from which the placental tissues were sourced, such as maternal age or blood pressure, placental or fetal weight at delivery, etc., which could have been useful for further analysis and correlations. Additionally, placental tissues were not able to be distinguished by primary or secondary Cesarean section or between early or late onset preeclampsia. Secondly, having received the placental tissues as paraffinized samples from the tissue repository, further marker validation for our findings such as via ELISA or Western Blot was unfortunately not possible. Additional investigation to validate these results is warranted.

Although further work is ultimately needed to fully characterize the upstream and downstream mechanisms involved in the HMGB1/RAGE/NFκB pathway leading to PE-associated inflammation within the placental tissue, the evidence reported in this paper supports the RAGE mechanistic pathway as a causal factor for this clinically impactful syndrome in pregnant women.

## Data Availability

The original contributions presented in the study are included in the article/Supplementary Material, further inquiries can be directed to the corresponding author.
