# Supplementary material for: Inflammatory mediators and the RAGE pathway in placental tissues of pregnancies complicated by severe preeclampsia
Source: Front Reprod Health. 2025 Aug 11;7:1587699. doi: 10.3389/frph.2025.1587699 (PMC12375582; doi:10.3389/frph.2025.1587699)
Supplement: Supplementary file 1 [file Table1.docx]

Supplementary Table S1:

IOD (Integrated Optical Density) of stained villi

|  | Negative Control | Positive Control | Pre-eclampsia |
| --- | --- | --- | --- |
| HMGB1 |  |  |  |
| Mean | 6223306.3 | 8010017.3* | 8300960.9* |
| ± SD | 1714148.8 | 3189795.4 | 1512379.2 |
| RAGE |  |  |  |
| Mean | 7726862.1 | 9704718.3* | 9657419.4* |
| ± SD | 1159520.9 | 1874112.3 | 2048728.7 |
| S100 |  |  |  |
| Mean | 6560906.5 | 8689740.9* | 7648124.3 |
| ± SD | 1463666.7 | 1493361.0 | 1447384.6 |
| COX2 |  |  |  |
| Mean | 6104099.1 | 7252114.4 | 6578763.9 |
| ± SD | 2064961.6 | 1908370.2 | 1478704.4 |
| IL8 |  |  |  |
| Mean | 3128248.8 | 4640467.8* | 4068320.1 |
| ± SD | 1362065.0 | 1977653.3 | 1836153.5 |
| NFkB |  |  |  |
| Mean | 5712046.1 | 8961492.5* | 8126427.2* |
| ± SD | 1522002.1 | 2487418.0 | 3380387.8 |

Mean ± SD of n=20/grp; ANOVA w/Tukey's multiple comparison test.

*Statistically significant to negative control
